# Supplementary material for: Mechanistic Insights into Substrate Recognition of Human Nucleoside Diphosphate Kinase C Based on Nucleotide-Induced Structural Changes
Source: Int J Mol Sci. 2024 Sep 10;25(18):9768. doi: 10.3390/ijms25189768 (PMC11431768; doi:10.3390/ijms25189768)
Supplement: Supplementary file 1 [file ijms-25-09768-s001.zip › ijms-3173776-supplementary.pdf]

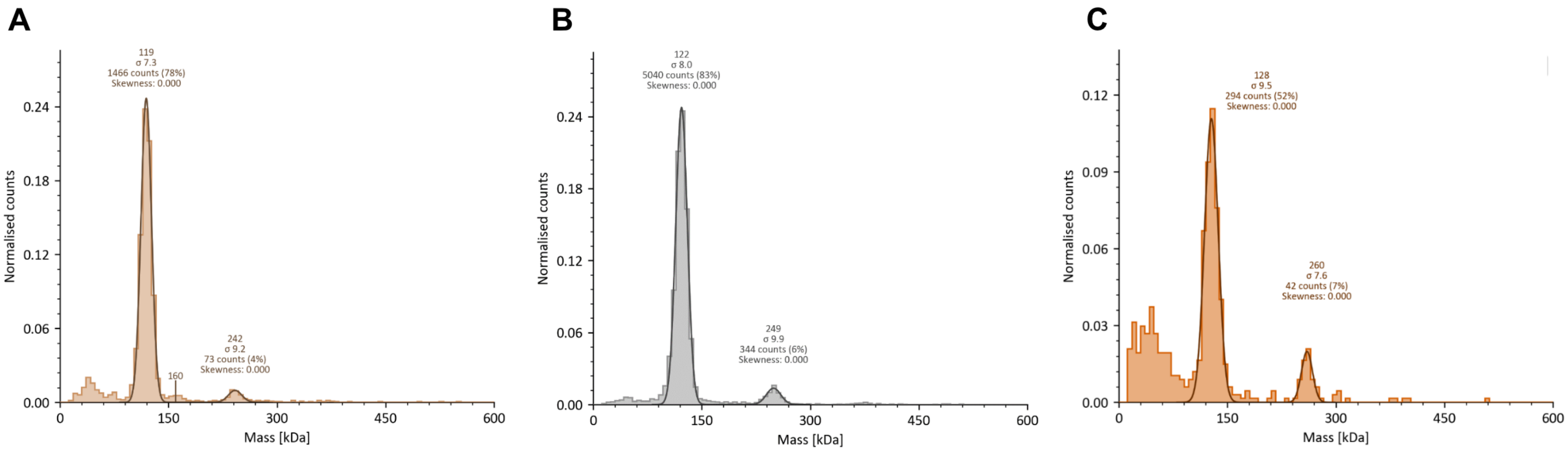

Supplemental Figure S1. *Recombinant His-tagged NDPK isoforms are hexamers in solution* - His-tagged human NDPK-A (A), NDPK-B (B) and NDPK-C (C) were expressed in E. coli and purified by affinity chromatography. Thereafter, the proteins were subjected to mass photometry at to detect oligomerization (Chen, H., et al, Curr Opin Struct Biol. 2021; 66: 112–118). The majority of all three isoforms were mainly detected in a single peak ranging between 119 – 128 kDa, which is in good agreement with the formation of a hexameric complex of the His-tagged NDPK proteins (calculated Mr ~ 120 kDa).

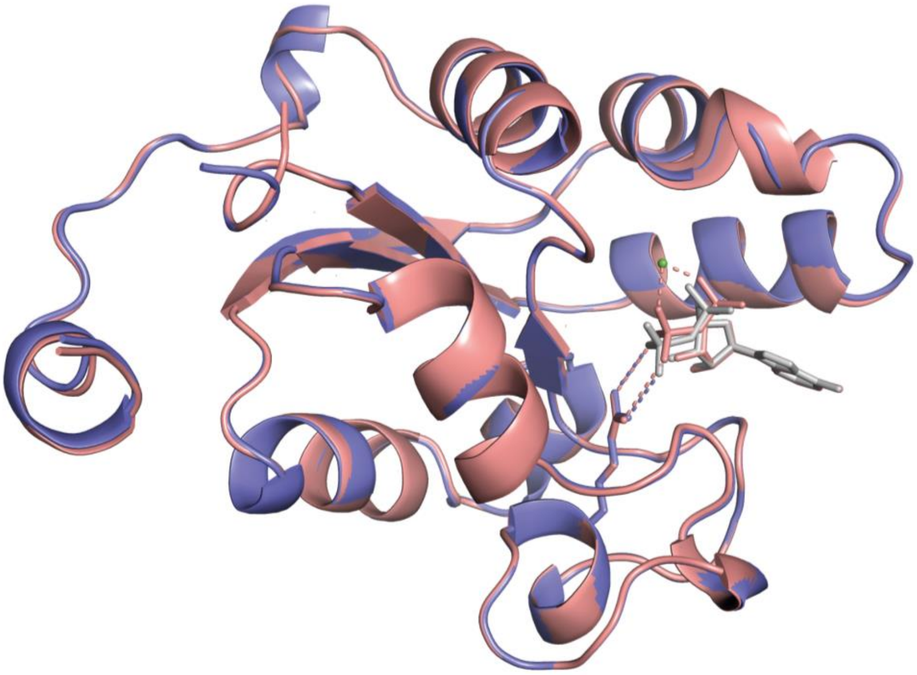

Supplemental Figure S2. *Comparison of overall conformation for the magnesium-free and magnesium-containing ADP complexes* - Super-position of the two structural models revealed for NDPK-C in the absence (blue) or presence (red) of magnesium ions. The over-all conformation of the protein is almost identical, but the localization of the nucleotide is different. The magnesium ion draw the beta phosphate group closer to the catalytically active histidine.

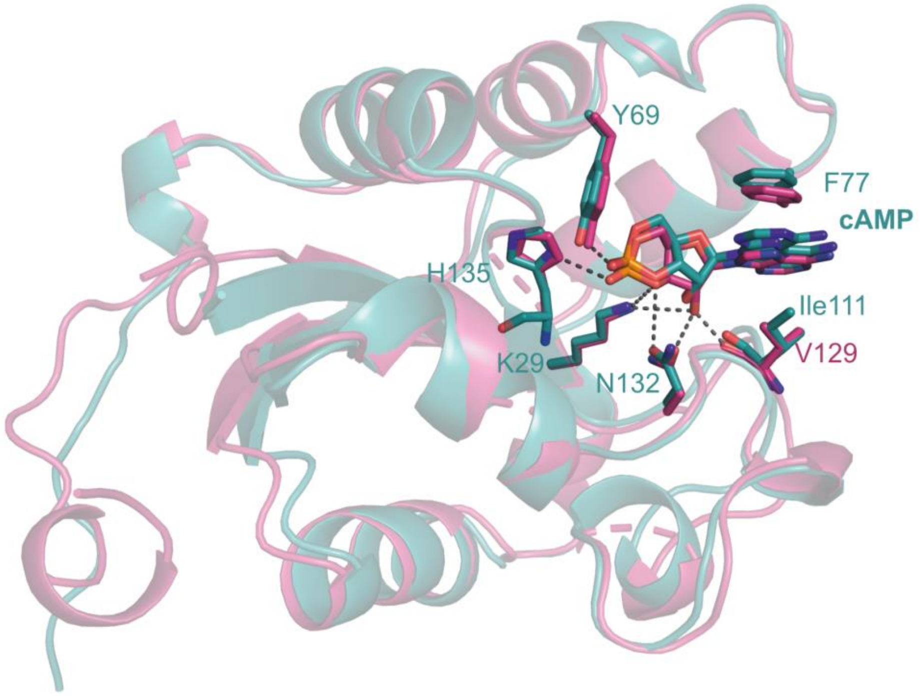

Supplemental Figure S3. *Comparison of bacterial and human cAMP complexes* - Superposition of cAMP complexes formed with H. sapiens NDPK-C (magenta) or M. xanthus NDPK (PDB entry 1NHK, shown in deepteal). The binding mode of the two proteins for cAMP is essentially identical.
